# *Usnea aurantiaco-atra* (Jacq) Bory: Metabolites and Biological Activities

**DOI:** 10.3390/molecules28217317

**Published:** 2023-10-28

**Authors:** María Jesús Vega-Bello, Mari Luz Moreno, Rossana Estellés-Leal, José Miguel Hernández-Andreu, Jesús A. Prieto-Ruiz

**Affiliations:** 1 Department of Basic Biomedical Sciences, Catholic University of Valencia “San Vicente Mártir”, C/Quevedo 2, 46001 Valencia, Spain; mj.vega@ucv.es (M.J.V.-B.); rossana.estelles@ucv.es (R.E.-L.); jesus.prieto@ucv.es (J.A.P.-R.); 2 Department of Anatomy and Physiology, Catholic University of Valencia “San Vicente Mártir”, C/Quevedo 2, 46001 Valencia, Spain; ml.moreno@ucv.es; 3 Molecular and Mitochondrial Medicine Research Group, Catholic University of Valencia “San Vicente Mártir”, 46001 Valencia, Spain

**Keywords:** antioxidant activity, cytotoxicity, phenolic metabolites, *Usnea aurantiaco-atra*, usnic acid

## Abstract

Background: Lichens are complex symbiotic associations between a fungus and an alga or cyanobacterium. Due to their great adaptability to the environment, they have managed to colonize many terrestrial habitats, presenting a worldwide distribution from the poles to the tropical regions and from the plains to the highest mountains. In the flora of the Antarctic region, lichens stand out due to their variety and development and are a potential source of new bioactive compounds. Methods: A phytochemical study of the Antarctic lichen *Usnea aurantiaco-atra* (Jacq) Bory was conducted with the intention of determining the most important metabolites. In addition, the cytotoxic and antioxidant activities of its extracts were determined. Results: Cytotoxicity studies revealed that the hexane extract contains usnic acid as a majority metabolite, in addition to linoleic acid, ergosterols and terpenes, and demonstrates cytotoxic activity against an A375 melanoma cell line. On the other hand, the presence of total phenols in the extracts did not influence their antioxidant activity. Conclusions: *U. aurantiaco-atra* contains mainly usnic acid, although there are terpenes and ergosta compounds that could be responsible for its cytotoxic activity. The presence of phenols did not confer antioxidant properties.

## 1. Introduction

Lichens represent one of the most successful examples of symbiosis between two different living beings, a fungus and one or more photosynthetic autotrophic organisms which can either be a green alga and/or a cyanobacterium [1]. Due to their great adaptability to the environment, they have managed to colonize many terrestrial habitats, presenting a worldwide distribution from the poles to the tropical regions and from the plains to the highest mountains. In the flora of the Antarctic region, lichens stand out due to their variety and development and are a potential source of new bioactive compounds [2].

More than 850 metabolites produced by lichens are currently known, which include aliphatic acids, derivatives of pulvinic acid, derivatives of hydroxybenzoic acid, depsides, depsidones, dibenzofuran derivatives, anthraquinones, naphthoquinones and phenolic compounds [3]. Many of these have shown various biological activities such as antibacterial, antiviral, antifungal, antitumor, analgesic, antipyretic and anti-inflammatory activities [4,5]. In addition, their great antioxidant capacities stand out since they can prevent the oxidative processes involved in various neurodegenerative pathologies, cancers, diabetes, Alzheimer’s disease and chronic inflammation [6,7].

The genus *Usnea (Parmeliaceae, Ascomycota)* consists of about 300 species distributed from polar to tropical areas [8]. It has several subgenera, with *Neuropogon* representing most of the Antarctic species. In this subgenus are four main species: *Usnea antarctica*, *Usnea aurantiaco-atra*, *Usnea sphacelata* and *Usnea subantarctica* [9]. The greatest abundance and diversity of species can be found in the Antarctic Peninsula [10]. *U. aurantiaco-atra* is present in the Falkland Islands, the southern tip of South America, the western coasts of the Antarctic Peninsula and in the sub-Antarctic islands, the Orkney Islands and the South Shetland Islands [9,10,11].

In the approximately 300 species that comprise the *Usnea* genus, the variety and amounts of metabolites present in them can be very different. It is known that their phytochemistry is very closely related to their external conditions and the environment in which they live, and that the former also influence their morphology [12]. Among the primary metabolites of this genus, it is possible to find proteins and amino acids; carotenes such as neoxanthin, antheraxanthin, zeoxanthin and lutein; polysaccharides such as raffinose, galactose, treolose, sucrose; and vitamins [13]. The group of secondary metabolites are more varied, among which there are depsides and depsidones, phenolic compounds such as orsellinic and β-orsellinic acid and many substances with an acid nature, such as lecanoric acid, fumarprotocetraric acid, protocetraic acid, norstictic acid and salicylic acid [14]. Terpenes stand out due to their importance and are found in small quantities; other substances found include fatty acids such as oleic acid, linoleic acid, myristic acid, hexadecanoic acid and palmitic acid [15].

Of all the secondary metabolites, the most typical and abundant compound in this genus is usnic acid, which is present in the cortex of each species, although in different amounts depending on the area of the thallus, which has traditionally been used to treat diarrhea, ulcers, urinary tract infections, tuberculosis, pneumonia, stomach pain and fungal diseases [15].

Our study complements other recent phytochemical analysis from the lichen *U. aurantiaco-atra* in which usnic acid, terpenes and ergosta compounds were present [16]. We aim to confirm the presence of these metabolites and to add new ones. As a novelty, we studied the antioxidant and cytotoxic activities of the lichen in other extracts that have not been described so far.

## 2. Results

### 2.1. Extraction Performance

Three extractions were made, using 30 g of lichen each with the aforementioned solvents. The results of the extractions performed for each solvent are shown in Table 1.

### 2.2. The Identification of Metabolites in Each Extract

#### 2.2.1. Hexane Extract

**Figure 1:**
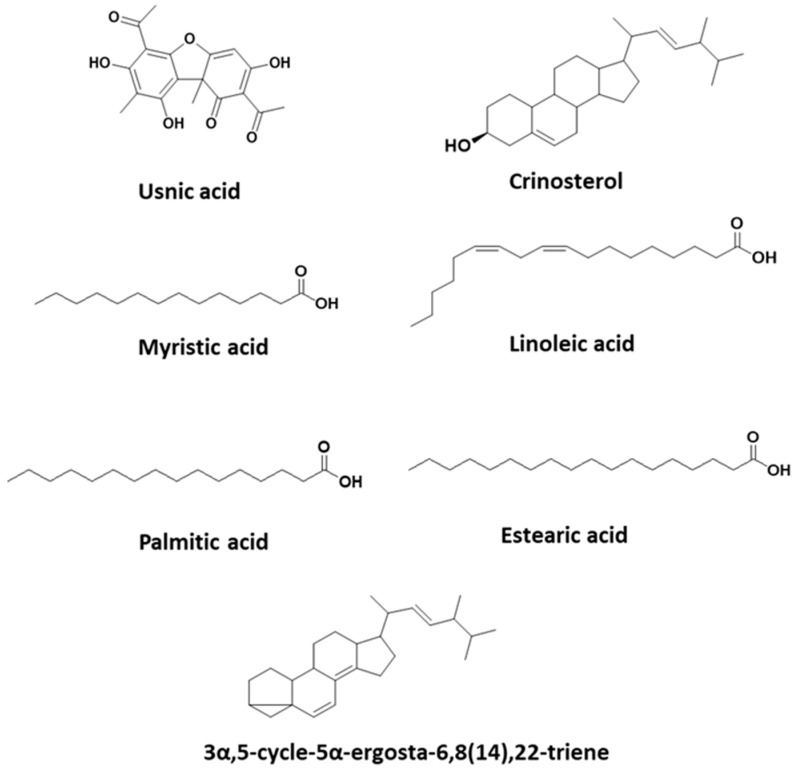
Chemical structures present in *U. aurantiaco-atra.*

The results show that the hexane extract has a high percentage of usnic acid (Table 2). Eight fractions numbered from F0 to F7 were obtained (Table 3). In addition, fractions F1 and F4 contain sterols and fatty acids. F4 is the only extraction that has sterols and a high percentage of linoleic acid. The chemical structures of usnic acid and other substances of interest present in the hexane extract are shown in Figure 1. The GC-MS spectra of usnic acid, crinosterol and 3α,5-cycle-5α-ergosta-6,8(14),22triene are shown in Supplementary Figures S1–S3.

The chromatograms of F1 and F4 are shown in Supplementary Figures S4 and S5.

#### 2.2.2. Dichloromethane Extract

Six fractions were obtained and numbered from F1 to F6 (Table 4). The fractions F3, F4, F5 and F6 contain usnic acid. The percentages in which it appears in each fraction are not as high as in the hexane extract. The amount of usnic acid present in the dichloromethane (DCM) extract is shown in Table 2. The chromatogram of F3 is shown in Supplementary Figure S6.

#### 2.2.3. Methanol Extract

Nine numbered fractions were obtained, ranging from F1 to F9 (Table 5). The fractions F4, F5, F6, F7, F8 and F9 contain usnic acid, although the percentages in which they appear are low. This is the extract that has usnic acid in lower percentages (Table 2). The metabolite proline-5-oxo-2-pyrrolidine carboxylate also appeared in F4 at a percentage of 48.61. This also appeared in the fractions F3 (5.44%), F5 (8.5%), F6 (3.29%), F8 (2.05%) and F9 (3.38%) and only in this extract. The F4 fraction also presents triterpenes, but being in such low proportions, they could not be identified via GC-MS. The presence of polyols, phenolic compounds and aromatic hydrocarbons stands out and could explain the significant amounts of total phenols, 68.61 ± 0.01 mg GA/mg, obtained in the assay for the determination of the total phenols. The chromatogram of F4 is shown in Supplementary Figure S7.

- The metabolites contained in each fraction of the derivatized methanol extract

Four fractions of different polarities were obtained, with SPE1 being the most polar. In this fraction, a high percentage of carbohydrates of four carbon atoms of the furanose type (T.R. 7.74 min) was found, among them ribitol (T.R. 7.9 min), which is a polyalcohol in the form of pentose that is formed via the reduction of ribose and other carbohydrates. The rest of the fractions of the derivatized methanol extract confirmed some of the compounds exposed in the table of the underivatized methanol extract.

### 2.3. Cytotoxic Activity

**Figure 2:**
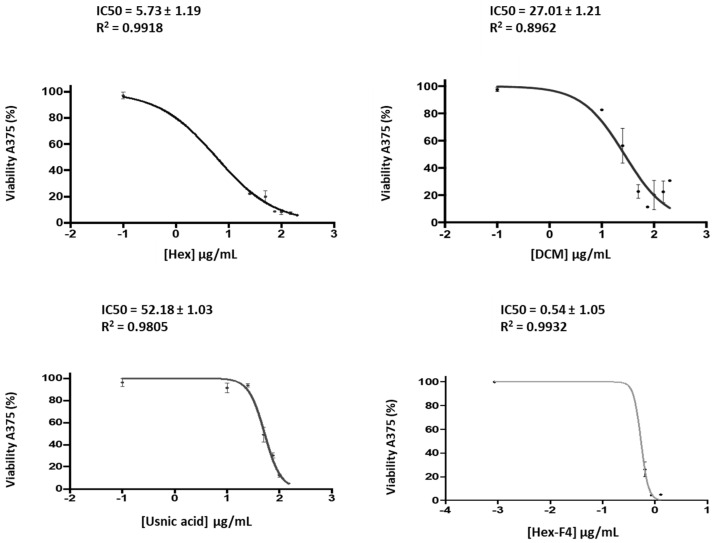
Cell viability of A375 after incubation with different concentrations of the hexane extract, DCM extract, usnic acid and fraction 4 from the hexane extract.

The results show (Figure 2) that Fraction 4 of the hexane extract is the one with the most cytotoxic activity, followed by the hexane and dichloromethane extracts and, finally, the methanol extract (MeOH) (Table 6). Since no negative controls were used, these results should be considered preliminary.

### 2.4. Antioxidant Activity

The IC50 of the methanol extract was 130.8 ± 4.3 μg/mL, and that of the BHT standard was 10.6 ± 3.7 μg/mL. Therefore, the IC50 of the methanolic extract is high, indicating that it has very little antioxidant capacity. According to Ramos and collaborators, a substance is considered to have a high antioxidant potential when its IC50 is below 30 µg/mL [17].

### 2.5. Total Phenolic Content (TPC)

The SP1, SP3 and SP4 fractions were eluted with water, methanol and ethyl acetate at 100%. The aim was to identify the fraction with the higher content of phenols but, as shown in Table 7, the results were very similar.

## 3. Discussion

### 3.1. Phytochemical Study

This is a complementary and additional phytochemical study of the lichen *U. aurantiaco-atra*, and it confirms that usnic acid is the main metabolite and is present in all three extracts, although in different amounts. Furthermore, the study shows that the levels of usnic acid found are much higher than those described in another species of the same genus such as *Usnea barbata*. This could be due to the location and climatic conditions in which the lichen develops [18,19].

Usnic acid is a lichen substance widely studied due to its many biological activities [20,21]. All this means that its majority presence in this lichen aroused our interest in researching it. In addition, other metabolites identified are ergosterols and terpenes, which are also relevant for their cytotoxic activity [22]. Among the ergosterols present, it was possible to identify 3α,5-cycle-5α-Ergosta-6,8(14),22-triene and Ergosta-5,22-dien-3-ol(3β.22Є.24Ѕ). Although ergosterols were found in other lichen genera, there is only one other study with *Usnea longissima* in which ergosterol compounds were also identified [23].

Regarding the first sterol, there is no specific scientific evidence of its biological activity, but it is known that many sterols are present in medicinal fungi, and some of them are used in traditional medicine due to their anti-aging properties, for their usefulness in treating neurodegenerative diseases such as Alzheimer’s [24] and for their anti-inflammatory and photoprotective activities [25] and their antitumor, cytotoxic and immunosuppressive activities [26,27]. The second sterol is commonly known as crinosterol, and it is present in seaweed and marine invertebrates [28,29,30,31]. It has been used in the treatment of Alzheimer’s due to its anti-aging properties and neuroprotective functions [32].

The reduced amount of starting lichen and the low efficiency obtained in the extractions made it difficult to identify other triterpenes and compounds. In addition, lichen metabolism is not sufficiently understood, and there are controversial opinions about the identification of various metabolites. According to Carlin [33], the chemical composition of any species of *Usnea* can depend on the metabolic state in which its thallus is found since many compounds that may seem different simply represent different oxidation states of the same substance [12].

The extracts also have high percentages of saturated and unsaturated fatty acids. These include linoleic acid, an unsaturated fatty acid that is attributed to different activities, among which its anticancer properties stand out [34,35]. Another metabolite that appeared in various fractions of the methanol extract was proline-5-oxo-2-pyrroldine carboxylate. Some plant studies also identified it in the methanol extract [36,37], and it has been noted that it is a non-essential amino acid related to collagen synthesis in humans. In addition, the methanol extract has ribose and abundant carbohydrates that coincide with other published data about *Usnea fasciata*, an Antarctic lichen whose species are very close to that of our study [38]. We also identified phenolic compounds in various fractions of the methanol extract in important percentages that are interesting because many studies claim that their presence is related to antioxidant activity [39,40].

### 3.2. Biological Activities: Cytotoxicity and Antioxidant Activity

Melanoma is related to skin exposure to UV light, and certain Antarctic organisms are known to have the ability to produce compounds that absorb UV radiation as a defense mechanism, thus surviving the high doses of UV radiation in Antarctica due to the ozone hole in the stratospheric layers [41]. Due to the extreme climatic conditions in which the lichen *U. aurantiaco-atra* lives, its secondary metabolites could have an interesting cytotoxic or antiproliferative activities on melanoma.

Usnic acid is a widely studied lichen metabolite, and is described as one of the 10 lichen substances with cytotoxic activity [42]. There are many studies that demonstrate this activity, such as those carried out in the cell lines FemX (melanoma) and LS174 (colon carcinoma) [43], MCF-7 (Breast adenocarcinoma) and HeLa (cervix adenocarcinoma) [44], A2780 (ovarian carcinoma) and HT-29 (colon adenocarcinoma) [45] and squamous cell carcinoma (A-431) [46]. In addition, studies were conducted on the cytotoxicity of *U. aurantiaco-atra* in methanol–acetone extracts that presented similar values as our methanol extract [47]. However, there have been no investigations on the cytotoxicity of *U. aurantiaco-atra* in DCM and hexane extracts. Our research shows that the hexane extract of this species is the one that contains the greater quantity of usnic acid and in which a greater cytotoxic activity can be observed. Other studies in which usnic acid from other lichens was studied observed cytotoxic activity very similar to ours [48,49], so it might be thought that the cytotoxic or antiproliferative activity of our extracts could be due to this metabolite. In addition, its amount in each of the extracts has a direct relationship with the IC50 value obtained in the melanoma cell line. However, when commercial usnic acid was added at the same concentration as in the extracts and under the same conditions, we observed that the IC50 values obtained were 10 times higher than those obtained with the extract. On the other hand, studies with lichens of the genus *Usnea* whose hexane extracts had high amounts of usnic acid showed low cytotoxicity rates. On the other hand, lichen species that did not have usnic acid in their hexane extracts were active against murine and human cell lines [42]. Therefore, our study suggests the existence of other compounds present in the extracts which differ from to usnic acid and could be responsible for the extracts’ cytotoxic activity.

Studies of other lichens that related cytotoxic activity with the presence of terpenes [27,28,50] were conducted. That is why we studied the cytotoxic activity of the F4 fraction of the hexane extract, which is the one with the highest percentages of terpenes and ergosterols; in addition, it contains a high percentage of linoleic acid and does not contain usnic acid. The IC50 obtained was approximately ten times lower than the IC50 obtained from the hexane extract. Thus, it cannot be confirmed that the activity of the hexane extract is due only to the presence of terpenes and crinosterol since it is difficult to determine in an extract the contribution of each of its components to the final activity, but a direct relationship was observed between the percentage of terpenes present in each extract and its cytotoxic or antiproliferative activity.

In summary, it can be said that the best results in terms of cytotoxicity were obtained with the hexane extract, and it can be considered that its extracts have in vitro cytotoxic activity against the A-375 skin cell line, as established by the National Cancer Institute of the United States (NCI), which states that a raw extract can be considered active when it has an IC50 ≤ 30 μg/mL [17]. The DCM extract also shows cytotoxic activity of interest. On the other hand, the cytotoxic activity of the methanol extract is low or null in the cell line tested. Therefore, our study shows that the hexane and dichloromethane extracts are more cytotoxic than usnic acid alone, which could be interpreted as the presence of a synergy between the different compounds that form the extracts.

Regarding the antioxidant activity of the extracts, Ramos and colleagues [51] proposed that a plant extract that has an IC50 value of lower than 30 μg/mL is considered to have high antioxidant potential, if the IC50 of the extract is between 30 and 100 μg/mL, its antioxidant potential has a moderate value, and an extract with an IC50 above 100 μg/mL is considered to have low antioxidant potential. According to this criterion, the antioxidant activity can be considered low. In general, a plant with a high content of total phenolic compounds has a high level antioxidant activity, though it can be observed in some cases that this correlation is not achieved [39,52]. This may be due to the fact that the antioxidant activity of a lichen is due to the development of synergies between its components or, on the contrary, that antagonistic interactions appear with other phenolic compounds present in the extract [53] or with other types of components such as carbohydrates and proteins [54], something that could happen in our study. In addition, in research studies which used other lichen species with phytochemistries similar to that of *U. aurantiaco-atra*, the IC50 values of antioxidant activity oscillate in a wide range of concentrations [55].

Numerous studies have shown that environmental factors play an important role in the antioxidant activities of lichens. Extreme environmental factors such as excessive radiation, desiccation and rehydration or pollution can decrease the synthesis of antioxidant substances [39,56,57]. Other authors suggest that the antioxidant activities of lichen extracts may not necessarily be correlated with polyphenolic components, suggesting that they may also depend on other non-phenolic lichen compounds [58]. Our study shows that the antioxidant activity of the methanol extract is not very high and that the cytotoxic activity of the A-375 line is very low. The methanol extract contains a low percentage of usnic acid, considered in some studies a compound with a good antioxidant activity, which could explain the low antioxidant activity of this extract [59].

### 3.3. Limitations

The limitations of this study include the small initial amount of lichen that we had for the study. Therefore, we tried to use as little as possible for each experiment. The reduced sample quantity especially affected the in vitro studies, forcing us to focus only on the melanoma cell line due to the localization of the lichen and its relationship with the ozone layer. Further studies are required to test our preliminary cytotoxicity results in healthy cells, as well as in other cancer cell lines.

## 4. Materials and Methods

### 4.1. Lichen Sample

The samples of the lichen *U. aurantiaco-atra* were collected in the vicinity of the Spanish Antarctic Base “Juan Carlos I”, which is located in the South Shetland Islands. Specialized Navy personnel and researchers present in Antarctica were in charge of collecting and identifying the lichen specimen object of our study. Since *U. aurantiaco-atra* is a protected species, it was necessary to comply with the Protocol of the Antarctic Treaty on the protection of the environment to ensure the preservation of native species. This protocol dictates that the passage through areas with concentrations of birds should be always avoided, and an area with an abundant distribution of this type of lichen should be chosen, carrying out a dispersed collection of the lichen in a way that allows for the easy repopulation of the lichen.

Once the samples were collected, they were transferred in BIO Hespérides to the Naval Base of Cartagena (Spain). The conservation and maintenance of the samples along the route was carried out in the cold, between 5 and 8 °C, applying sprayed irrigation using deionized water every 15 days. Once the samples arrived in Spain, they were transferred to the Biomedicine laboratory of the Catholic University of Valencia “San Vicente Mártir” where they were washed with deionized water and placed in a glass container at −80 °C until their subsequent use.

### 4.2. Sample Preparation and Obtaining Extracts

The thawed samples were crushed and pulverized using a grinder and subjected to a metabolite extraction process using a Soxhlet apparatus with successive solvents of different polarities. The extraction began with the most apolar solvents, such as n-hexane (analytical grade, Scharlau S.L., Barcelona, Spain); then, a solvent of intermediate polarity, such as dichloromethane (analytical grade, Scharlau S.L., Barcelona, Spain), and, finally, a polar solvent such as methanol (analytical grade, Scharlau S.L., Barcelona, Spain). The volume of solvent used was 600 mL for each extraction. The extraction time was 4 h for each solvent, and the size of the pulverized sample was 30 g.

The solvents were removed using a reduced-pressure rotavapor (Buchi, Labortechnik, Switzerland) to obtain three extracts: a hexane extract (Hex), a dichloromethane extract (DCM) and a methanol extract (MeOH).

### 4.3. Column Chromatography

Flash column chromatography utilizing silica gel 60 (0.040–0.063 mm) (Merck-Millipore, Darmstadt, Germany) was performed for each of the hexane, dichloromethane and methanol extracts. In the mobile phase, solvents such as n-hexane, ethyl acetate, dichloromethane and methanol were used, as well as mixtures thereof in different proportions.

For the Hex extract, a gradient from 100% hexane up to 100% ethyl acetate was used. For the DCM extract, a gradient from 100% hexane up to 100% ethyl acetate was used, and, in addition, to finish the column, a mixture of dichloromethane/methanol (95:5) was used. In the case of the MeOH extract, the mobile phase used was dichloromethane/methanol (90:10). With the Hex extract, a flow of approximately 3 mL/min was used. With the other two extracts, the flow was slower, especially with MeOH. The fractions obtained for each extract were gathered for qualitative monitoring via thin-layer chromatography and developed with UV light, using a 254 nm wavelength and sulfuric p-anisaldehyde.

Eight fractions were collected from the Hex extract sample used, six fractions were collected from the DCM extract sample and nine fractions were collected from the MeOH extract sample.

### 4.4. Preparing the Methanol Extract for GC-MS

#### 4.4.1. The Solid-Phase Extraction (SPE) of the Methanol Extract

A total of 100 mg of methanol extract was filtered through an ExtraBond C18 cartridge (1 g, 6 mL, Scharlau S.L., Barcelona, Spain), which was previously pre-conditioned with methanol (3 × 6 mL) and distilled water (5 × 6 mL). Once the sample was prepared, it was eluted using distilled water, obtaining the SPE1 fraction to collect the most polar compounds. Next, the sample was eluted with a mixture of methanol and water at 50% to obtain the SPE2 fraction. Then, the SPE3 fraction was obtained by adding MeOH at 100% to the column. Finally, the SPE4 fraction was obtained by eluting the sample with ethyl acetate at 100%. The four fractions were concentrated in a reduced-pressure rotavapor.

#### 4.4.2. The Derivatization of the Methanol Extract

The derivatives of the methanol extract (5 mg) were obtained by means of a two-step process involving a process of methoxymation–trimethylsilylation. For the methoxyimation reaction, 5 mg of methanol extract was weighed in an amber gas vial which was treated with 100 μL of a methoxyamine hydrochloride solution (20 mg/μL in pyridine) and stirred overnight at room temperature. Next, N,O-Bis (trimethylsilyl) trifluoro-acetamide BSTFA at 1% (100 μL) (supplied by Sigma-Aldrich Co. St Quentin Fallavier, France) was added as a silylating agent and stirred for 3 h at room temperature [60]. Then, the vial was redissolved in 1 mL of CH_2_Cl_2_ and analyzed via GC-MS.

### 4.5. Gas Mass Chromatography (GC-MS)

The three extracts obtained were subjected to GC-MS. The initial amounts were 0.130 g of the hexane extract, 0.069 g of the DCM extract and 0.3375 g of the MeOH extract.

The identification of the main metabolites of the hexane and dichloromethane extracts was carried out from samples without derivatization and, in the case of the methanolic extract, it was carried out without derivatization and derivatization (silylation) because it contained more polar metabolites.

In the identification and quantification process, a gas chromatograph (GC) connected to a mass spectrometer (GC-MS, Perkin Elmer Clarus 500 gas chromatograph, Shelton, CT, USA) was used, operating in electronic impact mode (ionization energy 70 eV and ionization source temperature of 300 °C) and equipped with a ZB-5 MS capillary column (Phenomenex, Torrance, CA, USA). The range of *m*/*z* was between 40 and 600. Helium was used as a carrier gas at a flow of 1 mL/min.

The derivatized samples were injected using an autosampler at a temperature of 250 °C, which injected 1 μL of sample into the column in split mode (ratio: 4:1, flow: 5 mL/min). The temperature ramp was as follows: it started at 110 °C, increased by 25 °C/min up to 210 °C, increased by 10 °C/min up to 260 °C and, finally, increased by 4 °C/min up to 340 °C, which was sustained for 5 min. The software used for the identification was TurboMassVer5.2.0 (PerkinElmer, Inc., Norwalk, CT, USA).

The different compounds were identified by comparing the time of retention (T.R.) and the fragmentation pattern obtained from the mass spectrometry chromatograms with standard samples of commercialized usnic acid via a comparison with the database of the GC-MS team (NIST). The percentage of area that each metabolite had was obtained as a function of the total area of the chromatogram and the name or type of metabolite shown via the GC-MS. Of all the metabolites detected via GC-MS, those in an area/total area were considered significant % ≥ at 5%. The values represent the average of the results obtained after analyzing the sample in triplicate via GC-MS.

### 4.6. The Quantification of Usnic Acid in the Extracts

A calibration line for usnic acid was produced in which dodecane (Sigma-Aldrich) was used as the internal standard and the usnic acid used as reference was (+)-usnic acid (98%), which was supplied by ALFA (AESAR) from the VWR laboratory GmbH& Co KG (Karlsruhe, Germany). Based on the usnic acid response provided via the GC-MS, two calibration lines were constructed for two different ranges of concentrations. The equations obtained were as follows:

- Line 1 (y = 0.0317x − 0.0035) with an R^2^ = 0.98 for low usnic acid concentrations (between 0.02 and 0.004 mg/mL).
- Line 2 (y = 0.1803x − 0.1676) with an R^2^ = 0.96 for higher usnic acid concentrations (between 0.6 and 0.02 mg/mL)

### 4.7. Cytotoxic Activity

The cell line used in this study were A-375 human melanoma cells (ATCC CRL-1619) from the American Tissue Culture Collection (Manassas, VA, USA). They were cultured in a high-glucose DMEM medium (Labclinics, Barcelona, Spain) supplemented with fetal bovine serum (FBS) at 10%, 1% non-essential NNAA amino acids (Sigma, Madrid, Spain) and 1% ATB (penicillin–streptomycin) (Sigma) at 37 °C under a humidified atmosphere of 95% air and 5% CO_2_. The cells were grown in T-75 (Fisher, Madrid, Spain) vials, and after several passes, they were seeded in 96-well-plates (Corning 3596 Costar Cluster Brand New/Nos, Madrid, Spain). The number of cells was determined via exclusion using Trypan Blue (Labclinics) and was 6400 cells per well 24 h before the addition of the extract and usnic acid (VWR Ref: H56612.06).

The hexane, dichloromethane and methanol extracts, Fraction 4 of the hexane extract and the commercial usnic acid were diluted in series in the cell medium. The IC50 values were calculated to assess the cytotoxic activity of the lichen. In addition, as the presence of terpenes and ergosta compounds in the extracts may be related to cytotoxicity, and knowing that the F4 fraction of the hexane extract was the richest fraction in these compounds and lacked usnic acid, it was added to the melanoma cells to determine the type of metabolite responsible for the activity.

The serial concentrations of the three extracts, F4 and usnic acid in a final volume of 200 μL per cell well plate were 150, 100, 75, 25 and 0.1 μg/mL, and eight repeats were performed for each concentration and a control consisting of cells without an extract. The incubation of the plates was carried out at 37 °C in an atmosphere of CO_2_ at 5% and 100% relative humidity, incubating each plate for 72 h.

The concentration of terpenes in F4 in a final volume of 200 μL per cell well plate can be seen in Table 8.

After 72 h of treatment, cell viability was determined via the MTT (3-[4,5-dimethylthiazole-2-yl]-2,5-diphenyltetrazolium bromide) reduction assay [61]. At the end of the treatment period, 10 μL of a solution of 5 mg/mL MTT filtered in PBS and 240 μL of the medium were added to the well plates, which were then incubated at 37 °C in 5% CO_2_ for 4 h. After this time, formazan crystals were dissolved using 150 μL of DMSO which was stirred for 5 min; then, metabolic activity was measured at 550 nm spectrophotometrically using a Perkin Elmer Victor X5 plate reader (TermoFischer: Madrid, Spain).

Cell proliferation was calculated as the absorbance ratio of the treated group divided by the absorbance of the control group multiplied by 100. The cytotoxic or antiproliferative effect was expressed as an IC50 (inhibitory dose inhibiting 50% of cell growth) value and by the magnitude of the maximum effect on the exposed cells. The IC50 values were calculated from the calibration curve using Prism 6 Gradpad software (Gradpad Software, Boston, MA, USA).

### 4.8. Antioxidant Activity: DPPH Free-Radical Scavenging Activity Assay

The ability to capture free radicals from the extracts was determined using the DPPH solution (Merck-Millipore) as a reference, following the method previously published by Fukumoto and Mazza [62] using BHT (2,6–di-t-butyl-4-methylphenol) (Sigma) as a reference. All reactions were carried out at room temperature and protected from light with aluminum foil and placed in a dark room for 30 min. After the time elapsed, spectrophotometric measurements were obtained at 520 nm (Victor X5, PerkinElmer). Equal amounts of DPPH and methanol were used as a standard and blank, respectively. The scavenging activity was calculated using the following formula:

Scavenging (%) = (Acontrol − Asample)/Acontrol × 100

where Asample is the absorbance of the test sample and Acontrol is the absorbance of the control.

The % inhibition of the methanol extract without derivatization and that of the BHT standard were represented against their respective concentrations to obtain two lines that allowed for the calculation of the IC50 values of the extract and the standard.

- The line corresponding to the methanol extract without derivatization was

Y = 25.22 X + 46.09 R^2^ = 0.982

- The line corresponding to the BHT standard was

Y = 586.5X + 43.79 R^2^ = 0.912

### 4.9. Total Phenolic Content (TPC)

The total phenol contents were determined using the methanol extract and the fractions derivatized from it. They were determined using a Folin–Ciocalteu reagent, according to the previously published method [63], using gallic acid as a reference.

A total of 2 mg of the extract was taken and dissolved in 50 mL of distilled water and stirred, preparing a mother solution from each sample. An aliquot of 0.5 mL was taken from the mother solution and mixed with 0.75 mL of Folin–Ciocalteu reagent. This was allowed to stand for 5 min at room temperature. Then, 0.75 mL of 20% sodium carbonate was added and stirred, leaving it to stand for 90 min in darkness at room temperature.

**Figure 3:**
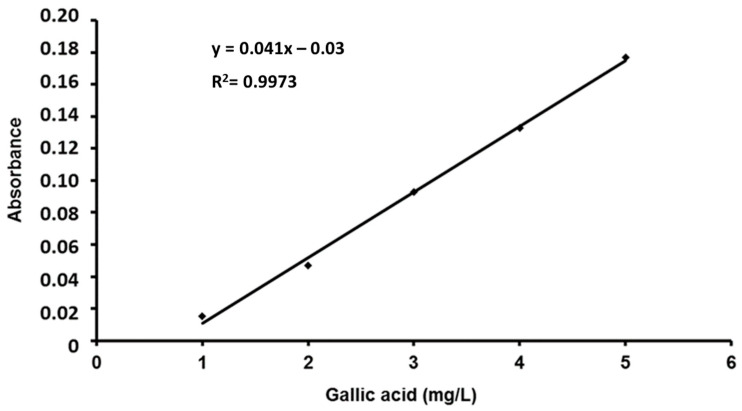
Gallic acid calibration line for the determination of total phenols.

A standard solution of gallic acid (Sigma-Aldrich), 0.1 g/L, was prepared. Then, a 1/10 dilution of gallic acid was prepared using distilled water. In the same way, a 20% solution of sodium carbonate (Sigma) was prepared. On the other hand, a 1 N solution of the Folin–Ciocalteu reagent (Sigma) was prepared. The reagent was protected from light and preserved in a refrigerator (4 °C) until its use. From the standard solution of gallic acid, the necessary dilutions were made to obtain concentrations of 1, 2, 3, 4 and 5 mg/L. The absorbance was read at 760 nm. All the experiments were performed in triplicate (Figure 3).

### 4.10. Statistical Analysis

All analyses were accomplished in triplicate, and the results are expressed as the means (n = 3) ± SDs, calculated using Microsoft 365 Office Excel (Redmond, Washington, DC, USA). The *p*-values were calculated using a one-way ANOVA, using R software (Version 4.0.5.). When the existence of differences between the study groups was determined, a Tukey post hoc analysis was applied in order to identify the groups with statistically significant differences (when the *p*-value was ≤0.05).

## 5. Conclusions

This is a phytochemical study of *U. aurantiaco-atra* that reports the presence of usnic acid in levels much higher than those described in the rest of the species. In addition, there are terpenes and ergosta compounds that could be responsible for its cytotoxic activity. The high concentration of usnic acid could respond to the adverse climatic conditions, as well as the presence of ergosta compounds. The presence of phenols did not confer antioxidant properties.

## Figures and Tables

**Table 1:** Extraction efficiency in different extracts.

Lichen (30 g)	Extraction Efficiency (g)
Hexane extract	1.04 ± 0.26 *
Dichloromethane extract	0.73 ± 0.15 *
Methanol extract	4.58 ± 1.02 *

Values are means ± SDs; n = 3. The efficiency values followed by * are statistically significant (*p* ≤ 0.05).

**Table 2:** Amounts of usnic acid present in extracts.

Extract	Usnic Acid in the Extract (mg)	Usnic Acid (µg)/Extract (mg)
Hexane 130 mg	109.81	844.66
Dichloromethane 69 mg	5.99	86.77
Methanol 337.5 mg	3.63	10.75

**Table 3:** Metabolites obtained from each fraction of the hexane extract.

HEXANE EXTRACT (130 mg)		AREA/TOTAL A.%							
T.R.	METABOLITE	F0 (83.8 mg)	F1 (5.5 mg)	F2 (5.6 mg)	F3 (4 mg)	F4 (8.38 mg)	F5 (3.2 mg)	F6 (12.5 mg)	F7 (5.2 mg)
5.31	2,4-decadienal		6.48	22.83					
7.65	Long-chain unsaturated hydrocarbon		17.22	2.08					
8.71	Unsaturated alcohol		9.37						
9.55	Hexadecanoic or Palmitic Acid					5.50	5.98		
10.80	Unsaturated hydrocarbon			5.41					
10.88	Linoleic acid					47.24	76.34		
11.00	Stearic acid					13.04			
11.04	Myristic acid						5.59		
12.70	Saturated long-chain acid		5.48	2.19					
12.79	Unsaturated hydrocarbon				12.05				
15.99	Unsaturated hydrocarbon		2.65						
**16.65**	**Usnic acid**	**99.45**						**95.54**	**86.3**
**17.55**	**3** **α** **,5-cycle-5** **α** **-ergosta-6,8(14),22-triene**		**7.52**						
**21.21**	**Ergosta-5,22-dien-3-ol (3** **β** **,22** **Є** **,24** **Ѕ** **)**					**8.08**			
**21.34**	**Triterpene**					**3.55**			
**21.51**	**Triterpene**					**1.79**			
28.05–28.07	Aromatic hydrocarbons		24.60	40.30	71.85	16.09	4.84	2.30	12.09
	**% TOTAL**	**99.45**	**73.32**	**72.80**	**83.90**	**95.29**	**92.76**	**97.84**	**98.34**

The percentage of area that each metabolite has was obtained as a function of the total area of the chromatogram and the name or type of metabolite shown via the GC-MS. Of all the metabolites detected via GC-MS, those in an area/total area were considered significant % ≥ at 5%. The values represent the average of the results obtained after analyzing the sample in triplicate via GC-MS. In some fractions, metabolites were indicated in low percentages (as is the case for terpenes) because they are considered of interest. Missing in each fraction until 100% can be completed are metabolites that were found in very low amounts. The main compounds found are highlighted in bold. T.R.: time of retention.

**Table 4:** Metabolites obtained in each fraction of the dichloromethane extract.

DICHLOROMETHANE EXTRACT (69 mg)		AREA/TOTAL A.%					
T.R.	METABOLITE	F1 (4.7 mg)	F2 (1.8 mg)	F3 (4.3 mg)	F4 (37.4 mg)	F5 (6.1 mg)	F6 (4.4 mg)
5.06	2-Decennial		6.32				
5.55	Unsaturated ester-type hydrocarbon					3.94	6.07
8.38	Unsaturated ester-type hydrocarbon					12.04	15.14
9.52	**Palmitic acid**		16.08	5.82			
10.05	Unsaturated alcohol					6.82	7.29
10.40	Unsaturated hydrocarbon					5.01	
10.42	Oleantrile	13.68					
10.97	**Stearic acid**			3.78			
11.16	**Oxalic acid**	5.02					
11.38	Aromatic alcohol-type compound					3.16	3.24
**16.45**	**Usnic acid**			**75.64**	**97.83**	**32.27**	**31.01**
17.13	Ester-type aromatic compound	30.85					
**21.17**	**Ergosta-5,22-dien-3-ol(3** **β** **.22** **Є** **.24** **Ѕ** **)**			**3.58**			
**21.33**	**Triterpene**			**1.15**			**2.3**
28.1	Aromatic hydrocarbons	45.43	54.17	2.56		26.72	27.38
	**TOTAL %**	**95**	**76.57**	**92.52**	**97.83**	**89.96**	**92.43**

The percentage of area that each metabolite has was obtained as a function of the total area of the chromatogram and the name or type of metabolite shown via the GC-MS. Of all the metabolites detected via GC-MS, those in an area/total area were considered significant % ≥ at 5%. The values represent the average of the results obtained after analyzing the sample in triplicate via GC-MS. The main compounds found are highlighted in bold. T.R.: time of retention.

**Table 5:** Metabolites obtained in each fraction of the methanol extract without derivatization.

METHANOL EXTRACT WITHOUT DERIV. (337.5 mg)		AREA/TOTAL A.%								
T.R.	METABOLITE	F1 (8 mg)	F2 (2.4 mg)	F3 (7.4 mg)	F4 (13.8 mg)	F5 (72 mg)	F6 (2.5 mg)	F7 (10.3 mg)	F8 (5 mg)	F9 (23.1 mg)
3.88	**Succinic acid**					3.92				1.38
3.93	**Fumaric acid**							2.12	13.11	2.09
5.06	2-Decennial				5.10					
5.59	Unsaturated alcohol					11.48				
5.86	Proline-5-oxo-2-pyrrolidine carboxilate			5.44	48.61	8.50	3.29		2.05	3.38
7.54	Polyalcohol								6.07	
7.82	Phenolic Compound									5.01
8.39	Long-chain hydrocarbon						1.31	2.90		17.83
8.66	Polyalcohol									18.53
9.53	**Palmitic acid**			1.40	7.59					
10.43	**Oxalic acid**			5.03						
10.51	**Linoleic acid**	**13.27**	**10.41**		**6.13**					
10.55	**Methylester linoleic acid**		11.88							
10.57	**Stearic acid**				9.3					
10.94	**Stearic acid**			5.01						
11.02	Alcohol-type unsaturated hydrocarbon			5.01	5.01					
13.05	Phenolic compound					5.64				
**16.15**	**Usnic acid**				**3.11**	**0.60**	**0.41**	**3.00**	**16.12**	**3.61**
**21.17**	**Triterpene**				**1.02**					
21.31	Unsaturated alcohol				0.55					
**21.93**	**Triterpene**				**0.62**					
24.60	Phenolic compound						23.47	29.07	3.39	11.02
28.25	Aromatic hydrocarbons	65.64	63.61	68.29	5.92	48.64	58.68	46.59	37.19	13.83
	**TOTAL %**	**78.91**	**85.90**	**90.18**	**92.96**	**78.78**	**87.16**	**83.68**	**77.94**	**76.68**

The percentage of area that each metabolite has was obtained as a function of the total area of the chromatogram and the name or type of metabolite shown via the GC-MS. Of all the metabolites detected via GC-MS, those in an area/total area were considered significant % ≥ at 5%. The values represent the average of the results obtained after analyzing the sample in triplicate via GC-MS. The main compounds found are highlighted in bold. T.R.: time of retention.

**Table 6:** The IC50 values of hexane, dichlorometane, the methanol extracts, Fraction 4 of the hexane extract and usnic acid in an A375 IC50 melanoma cell line.

	IC50 µg/mL *
Hexane Extract	**5.73 ± 1.19**
F4 of Hexane Extract	**0.54 ± 1.05**
DCM Extract	**27.02 ± 1.21**
MeOH Extract	293.3 ± 1.17
Usnic Acid	**52.18 ± 1.03**

* Each value in the table was obtained by calculating the average of three analyses ± the standard deviation. The best results of IC50 are highlighted in bold

**Table 7:** TPCs of the underivatized and derivatized MeOH extracts.

EXTRACT	TPC (Gallic Acid mg/g of Extract) *
MeOH without derivatization	68.61 ± 0.01
MeOH SP1 (Water 100%)	64.28 ± 0.02
MeOH SP3 (MeOH 100%)	67.15 ± 0.02
MeOH SP4 (AcOEt 100%)	66.92 ± 0.03

Total phenolic contents expressed as the gallic acid equivalent (mg GA/g of extract). * Each value in the table was obtained by calculating the average of three analyses ± the standard deviation.

**Table 8:** Serial equivalence between the total concentration of the hexane extract and the concentration of terpenes in F4 per well plate.

Concentration of hexane extract in each cell well plate	150 μg/mL	100 μg/mL	75 μg/mL	25 μg/mL	0.1 μg/ml
Corresponding terpene concentration in F4 in each well plate	1.29 μg/mL	0.86 μg/mL	0.64 μg/mL	0.29 μg/mL	8.6 × 10^−4^ μg/mL

## Data Availability

All data generated or analyzed during this study are included in this published article and its Supplementary Materials files.

## Supplementary Materials

The following supporting information can be downloaded at https://www.mdpi.com/article/10.3390/molecules28217317/s1, Figure S1: GC-MS spectrum of usnic acid; Figure S2: GC-MS spectrum of crinosterol; Figure S3: GC-MS spectrum of 3α,5-cycle-5α-ergosta-6,8(14),22-triene; Figure S4: Chromatogram of F1 from the hexane extract; Figure S5: Chromatogram of F4 from the hexane extract; Figure S6: Chromatogram of F3 from the DCM extract; Figure S7: Chromatogram of F4 from the MeOH extract.
