# Supplementary material for: Usnea aurantiaco-atra (Jacq) Bory: Metabolites and Biological Activities
Source: Molecules. 2023 Oct 28;28(21):7317. doi: 10.3390/molecules28217317 (PMC10649588; doi:10.3390/molecules28217317)

Usnea aurantiaco-atra(Jacq) Bory: metabolites and biological activities

Journal name: Molecules

María Jesús Vega-Bello, Mari Luz Moreno, Rossana Estellés-Leal, José Miguel Hernández-Andreu*, Jesús A Prieto-Ruiz

***Correspondence**

José Miguel Hernández Andreu

Department of Basic Biomedical Sciences. Catholic University of Valencia "San Vicente Mártir", C/Quevedo 2, 46001 Valencia, Spain

Molecular and Mitochondrial Medicine Research Group, Catholic University of Valencia "San Vicente Mártir", 46001 Valencia, Spain

E-mail: jmiguel.hernandez@ucv.es

Figure S4. Chromatograme of F1 from the hexane extract

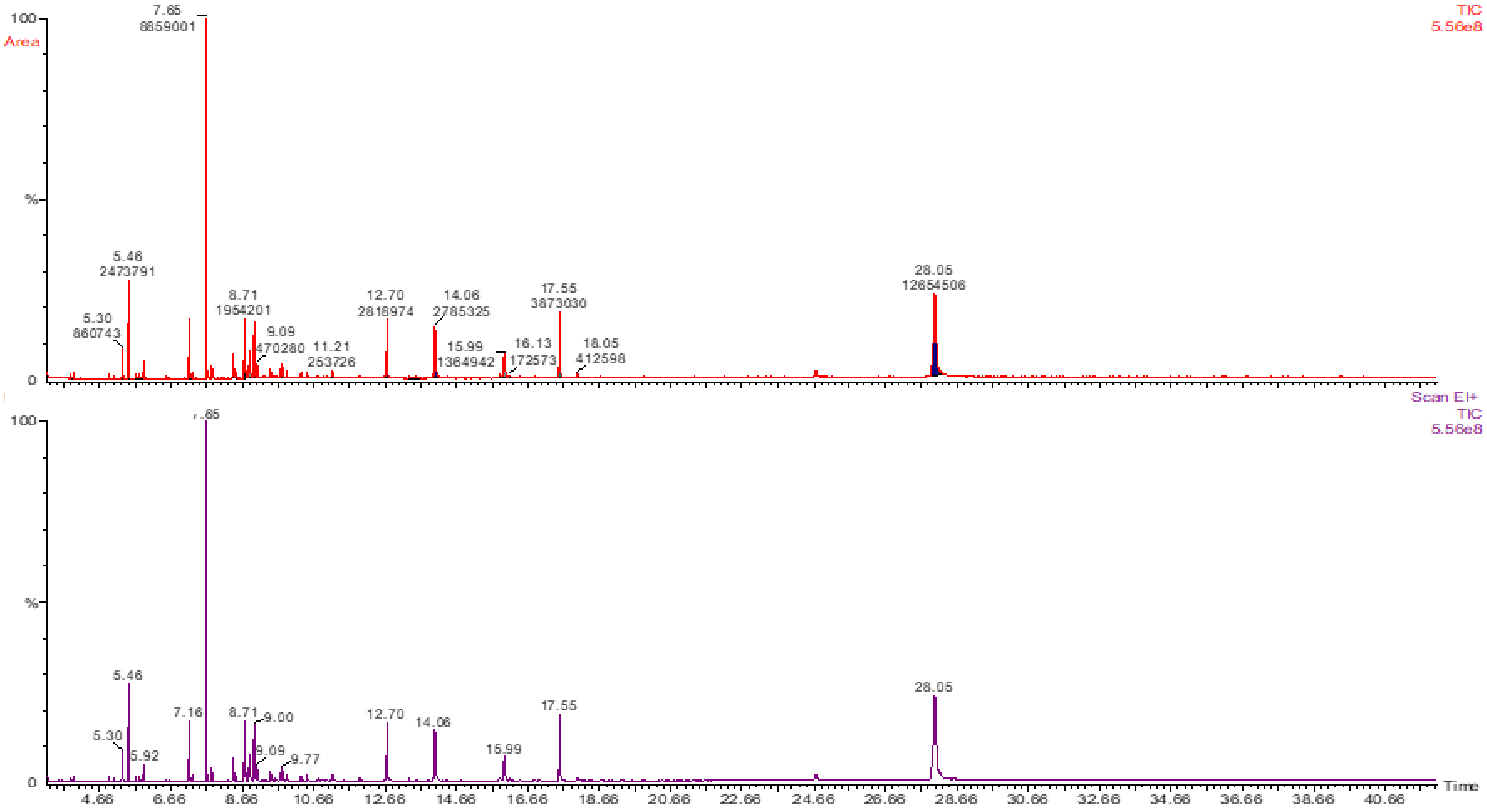

Supplement: Supplementary file 1 [file molecules-28-07317-s001.zip › Supplemmentary Figure S4.pdf]
